# The Effect of *Ocimum basilicum* L. and Its Main Ingredients on Respiratory Disorders: An Experimental, Preclinical, and Clinical Review

**DOI:** 10.3389/fphar.2021.805391

**Published:** 2022-01-03

**Authors:** Ahmad Reza Aminian, Reza Mohebbati, Mohammad Hossein Boskabady

**Affiliations:** ^1^ Department of Physiology, Faculty of Medicine, Mashhad University of Medical Sciences, Mashhad, Iran; ^2^ Applied Biomedical Research Center, Mashhad University of Medical Sciences, Mashhad, Iran

**Keywords:** *Ocimum basilicum* L., lung, linalool, respiratory disorders, extract

## Abstract

*Ocimum basilicum* L. (*O. basilicum*) and its constituents show anti-inflammatory, immunomodulatory, and antioxidant effects. The plant has been mainly utilized in traditional medicine for the treatment of respiratory disorders. In the present article, effects of *O. basilicum* and its main constituents on respiratory disorders, assessed by experimental and clinical studies, were reviewed. Relevant studies were searched in PubMed, Science Direct, Medline, and Embase databases using relevant keywords including “Ocimum basilicum,” “basilicums,” “linalool,” “respiratory disease,” “asthma,” “obstructive pulmonary disease,” “bronchodilatory,” “bronchitis,” “lung cancer,” and “pulmonary fibrosis,” and other related keywords.The reviewed articles showed both relieving and preventing effects of the plant and its ingredients on obstructive pulmonary diseases such as chronic obstructive pulmonary disease (COPD), asthma, and other respiratory disorders such as bronchitis, aspergillosis tuberculosis, and lung cancer. The results of the reviewed articles suggest the therapeutic potential of *O. basilicum* and its constituent, linalool, on respiratory disorders.

## Introduction

Respiratory diseases mainly include chronic obstructive pulmonary disease (COPD), asthma, pneumonia, pulmonary fibrosis, and lung cancer (Ferkol and Schraufnagel, 2014). These diseases affect a large number of people every year and reduce a person’s level of performance in daily activities (Zar and Ferkol, 2014), and they are the most common cause of referral to general practitioners worldwide (Chou et al., 2012). The rate of respiratory dysfunction due to pulmonary diseases depends on the disease type and severity (Bellamy et al., 2006; Pahal and Sharma, 2019). In the last decades, environmental agents such as pollutants and diet as well as individual factors such as genetics and epigenetics resulted in a rise in the prevalence of inflammatory, allergic, malignancy, and immunodeficiency disorders (Gea et al., 2015). These factors may result in a cascade of destructive and inflammatory mechanisms creating the pathological symptom of allergic diseases including asthma (Balkissoon et al., 2011). Early diagnosis, treatment, prevention, and monitoring of the respiratory disorders as well as the development of new therapeutics to alleviate such pathological mechanisms and restore the balance of the immune system are required for curing respiratory diseases (Gholamnezhad et al., 2015). Inflammatory obstructive respiratory diseases are treated mainly by two types of drugs, including relieving drugs that reduce airway obstruction and preventive drugs that reduce lung inflammation (Rabe and Schmidt, 2001).

Currently, two types of drugs are used to relieve symptoms and prevent the progression of the disease severity in various respiratory disorders such as asthma and COPDs. The relieving drugs used for the treatment of asthma are beta-2 agonists, anticholinergics, methylxanthines, corticosteroids (mainly inhaled forms), leukotriene modifiers, mast cell stabilizers, and immunomodulators (Mullane, 2011). Various immunomodulators are used for the treatment of respiratory diseases, including omalizumab, an anti-IgE antibody; benralizumab, mepolizumab, and reslizumab, IL-5 antibodies; and dupilumab, an IL-4 and IL-13 inhibitor monoclonal antibody which is used for the treatment of allergic asthma; some other antibodies are used for the treatment of other respiratory disorders such as lung infection and lung cancer (Desoubeaux et al., 2016).

**FIGURE 1:**
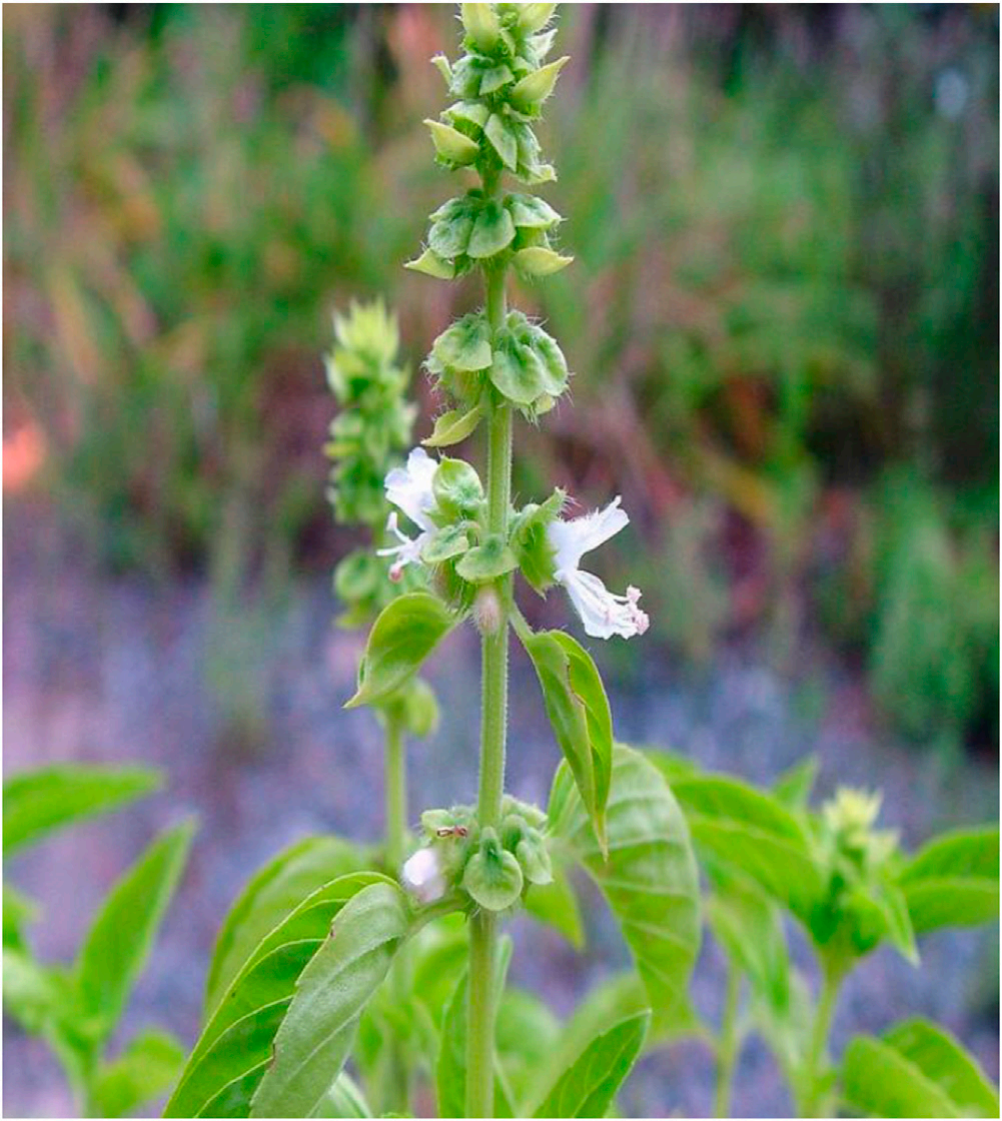
Natural view of *Ocimum basilicum*.

However, drugs that are currently used for the treatment of respiratory diseases lack full therapeutic efficacy and show major side effects. Therefore, the development of new drugs with higher efficacy and fewer side effects for the treatment of respiratory disorders is needed (Mullane, 2011). Herbal medicines are safe agents that are used as alternative and complementary therapeutics. *Ocimum basilicum* L. (*O. basilicum*) or basil is an important essential oil crop, medicinal plant, and culinary herb that belongs to the Lamiaceae family (Figure 1); it is an herbal plant wildly cultivated in regions of Central and Southeast Asia such as Iran and Pakistan (Sajjadi, 2006; Gholamnezhad et al., 2019). *O. basilicum* is a well-known herb traditionally used in different cultures across the globe for the management of lung diseases and other organ disorders (Boskabady et al., 2006).

*O. basilicum* is used to manage lung disorders and dyspnea in Iran (Noumi, 2009); asthma in Cameroon (Nath et al., 2008); bronchitis, cough, and asthma in India (Lemos et al., 2016); acute lung diseases, including bronchitis, cough, and sore throat, in Brazil (Janbaz et al., 2014); and tuberculosis and acute lung diseases in Ethiopia (Fujita et al., 1999). The bioactive ingredients of the plant depend on the growth regions (Driesen et al., 2021; Khater et al., 2021). The effects of *O. basilicum* and its derivatives on dermal pathology and wound healing including acne, eczema, boils, psoriasis, and rashes were reported (Fritea et al., 2021).

*O. basilicum* is usually used for the treatment of some disorders related to the respiratory tract including asthma, bronchitis, cough, gastrointestinal disorders (Othman et al., 2021; Touiss et al., 2021), cardiovascular diseases (Janbaz et al., 2014; Abidoye et al., 2022), neurocognitive disorders (Heshami et al., 2021), and metabolic disorders (Kiyose et al., 2021).

**FIGURE 2:**
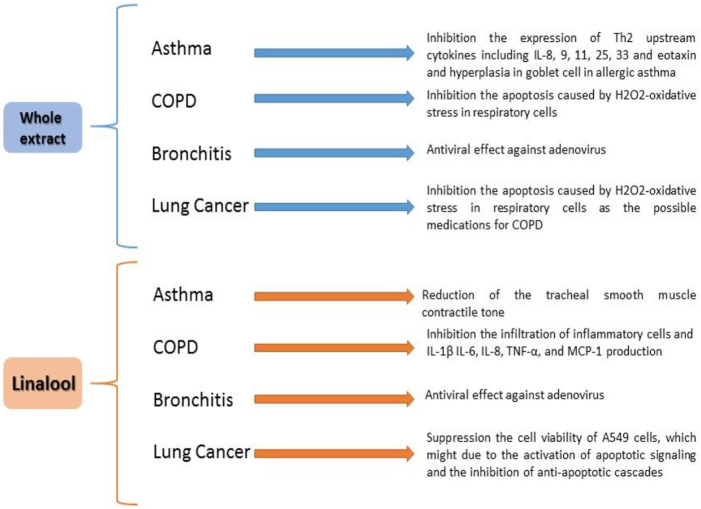
Effects of *Ocimum basilicum* and linalool on respiratory disease.

Several pharmacological impacts have been shown for *O. basilicum*, including anti-inflammatory (Neamati et al., 2016; Złotek et al., 2016; Saeidi et al., 2018), antioxidant (Eftekhar et al., 2018a; Teofilović et al., 2021), and bronchodilatory properties (Janbaz et al., 2014; Figure 2).

*O. basilicum* flowers and leaves are consumed in the form of infusion, syrup, decoction, as a sudorific, stimulant, carminative, diuretic, and febrifuge agent, and are frequently suggested for bronchitis and coughs (Vieira and Simon, 2000).

*O. basilicum* leaves have a strong antioxidant effect due to the existence of some constituents such as eugenol and vicenin (Ziaei et al., 2014). In addition, this plant showed anti-inflammatory properties due to the presence of citronellol, limonene, and eugenol in its leaf (Ziaei et al., 2014).

**FIGURE 3:**
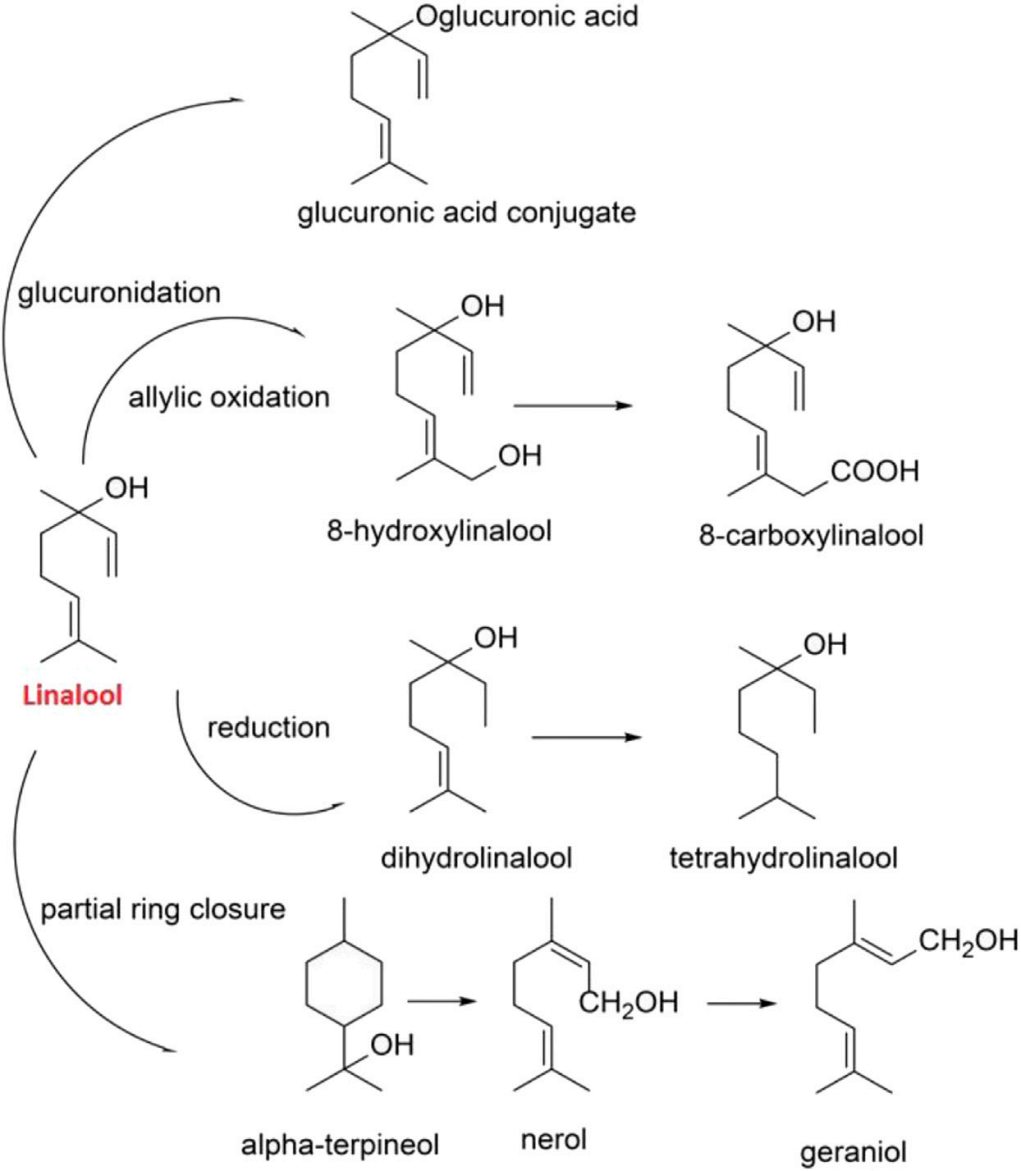
Linalool molecular structure and metabolites.

Phytochemical analysis and pharmacological studies showed that linalool is the main constituent of *O. basilicum* concerning both the amount and pharmacological activities of the plant (Rezzoug et al., 2019). Linalool metabolites are excreted in urine as free forms or conjugates. Products of linalool reduction (dihydro- and tetrahydrolinalool) were identified in rodent urine. Some of the bronchodilatory properties of this plant are mainly related to linalool metabolites such as alpha-terpineol, nerol, and geraniol. A significant proportion of orally administered linalool follows intermediary metabolic pathways as shown in Figure 3 (Elsharif et al., 2015).

The aim of the current article was to review experimental and clinical effects of *O. basilicum* and its main constituent, linalool, on respiratory disorders.

## Materials and Methods

Appropriate experimental and clinical studies were searched in PubMed, Science Direct, Medline, and Embase databases using relevant keywords including “*Ocimum*,” “*Ocimum basilicum*,” “*basilicums*,” “linalool,”, “respiratory disease,” “asthma,” “obstructive pulmonary disease,” “bronchodilatory,” “bronchitis,” “lung cancer,” and “pulmonary fibrosis,” and other related keywords published from 1989 until September 2020. The searched keywords were selected based on the MeSH alone and combined. The reviewed articles were available in full-text and written in English in well-recognized journals.

## Results

### The Main Ingredients of *O. basilicum*

*O. basilicum* has more than 100 bioactive ingredients including various vitamins, electrolytes, minerals, and phytonutrients (Marwat et al., 2011; Da Costa et al., 2015). The chemical compositions of essential oils (Özcan and Chalchat, 2002) of *O. basilicum* include methyl chavicol (estragole), cineole, eugenol, methyl eugenol, elemicin, myristicin, rosmarinic acid, linalool, apigenin, ursolic acid, and methyl cinnamate (Mullane, 2011; Amer et al., 2021; Senthoorraja et al., 2021).

Also, monoterpenes (ocimene, geraniol, and camphor), sesquiterpenes (bisabolene and caryophyllene), and phenylpropanoids (methyl cinnamate and methyl eugenol) are present in varying amounts and strongly influence the flavor of the plant. Linalool is a monoterpenoid which is octa-1, 6-diene substituted by methyl groups at positions 3 and 7, and a hydroxyl group at position 3 (Letizia et al., 2003). The presence of anthocyanins, anthraquinones, tannins, reducing sugars, glycosides, proteins, amino acids, flavonoids, and volatile oils, and the non-appearance of terpinoids and alkaloids were demonstrated as constituents of *O. basilicum* as well as the presence of steroids in an aqueous extract of the plant. Also, it was observed that the antioxidants and anticancer compounds are higher in the aqueous extract of the plant (Alkhateeb et al., 2021). Various chemical and physical conditions could influence the constituents of the plant. The promotion of the stress condition of metals, aluminum, lead, and cadmium in *O. basilicum* resulted in the reduction of the dry matter mass and increased the synthesis of phenolic compounds (Do Prado et al., 2022). The rosmarinic acid (RA) content of *O. basilicum* also increased due to the application of chitosan lactate (Hawrylak-Nowak et al., 2021). In addition, applications of aromatic amino acid composition reduced the expression of the *TAT* gene in the leaves, and the contents of chicoric acid, methyl chavicol, caffeic acid, and vanillic acid were increased (Kisa et al., 2021). The hairy root production increased in *O. basilicum* (green basil “Cinnamon”) when it was cultured under the light but it increased in purple basil (Purpurascens) when it was cultured under dark conditions. However, the content of RA in the cultured hairy roots of green basil was higher than that of purple basil under both light and dark conditions (Kwon et al., 2021). It was shown that excess zinc decreased chlorophyll content, total phenol and total flavonoid contents, as well as TBARS levels, which indicate an oxidative burst in *O. basilicum* (Mahmoudi et al., 2021). A study also indicated that *O. basilicum* grown on media supplemented with copper oxide nanoparticles (CuO-NPs) increased phenolic contents, flavonoid contents, and antioxidant activities compared to manganese oxide nanoparticles (MnO-NPs) and control. Superoxide dismutase (SOD) and peroxidase (POD) activities were also higher in CuO-NP cultures than in MnO-NPs and control. A negative impact of contaminated soil with cadmium (Cd) and lead (Pb) on morphological trait development of basil was shown. Cultivated plants with Cd- and Pb-contaminated soils showed a major influence on the EOs’ yield, composition, and phytoremediation of the soil (Youssef, 2021). In addition, RA, chicoric acid, and eugenol were higher when cultured in CuO-NP media (Nazir et al., 2021). Increase salicylic acid and its β-glucoside cause to accumulation of jasmonic and abscisic acids in *O. basilicum* due to foliar-spraying treatment with *U. intestinalis* extract and the essential oil (EO) chemotype changed from methyl eugenol/eugenol to epi-α-cadinol and increased sesquiterpenes (Paulert et al., 2021). Table 1 summarizes different constituents of *O. basilicum*.

**TABLE 1:** Chemical composition of *O. basilicum*.

Part of plant	Major constituent	%	Reference
Seed	Linalool	31.6	Stanojevic et al. (2017)
	Chavicol	23.8	
	Linalool	35.99	Mehdizadeh et al. (2016)
	1,8-cineole	22.91	
	p-Cymene	35.5	
	Eugenol	50.8	Mota et al. (2020)
	Linalool	54.7	
Leaf	Linalool	52.1	Rezzoug et al. (2019)
	Linalyl acetate	19.1	
	Methyl chavicol	47	Kavoosi and Amirghofran (2017)
	Geranial	19	
	Neral	15	
	Methyl eugenol	42.18	Kumar et al. (2011)
	Eugenol	4.89	
	1,8-cineole	4.88	
	B-Caryophyllene	4.37	
	Estragole	87.869	Tran et al. (2018)
	Cadinol	2.922	
	α-Bergamotene	2.770	
	τ-Linalool	1.347	
	Linalool	29.23	Chaaban et al. (2019)
	Methyl cinnamate	18.97	
	Eugenol	5.84	
	Eugenol	42.74	El Mokhtari et al. (2019)
	Linalool	20.54	
	Eucalyptol	15.27	
	Estragole	55.95	Elsherbiny et al. (2016)
	1,8-Cineole	10.56	
	Methyl eugenol	10.09	
	Linalool	5.57	
	β-Guaiene	16.89	Sundararajan et al. (2018)
	Cadinol	15.66	
	Nona-2, 4, 6-triene	11.36	
	Phytol	11.68	
	Methyl-chavicol	52.4	Venancio et al. (2011)
	Linalool	20.1	
	Epi-a-cadinol	5.9	
	Trans-a-bergamotene	5.2	
Overground part	Methyl eugenol	78.02	Özcan and Chalchat (2002)
	α-Cubebene	6.17	
	Nerol	0.83	
	ε-Muurolene	0.74	
	4,7-Dimethoxy-1-indanone	21.73	Kadan et al. (2016)
	Palmitic acid	7.60	
Flower	Linalool	72.3	Bobakulov et al. (2020)
	Methyl chavicol	19.5	

### The Effects of *O. basilicum* and Its Main Constituent on Respiratory Disorders: Experimental Evidence

#### Relaxant Effect of *O. basilicum* and Its Main Constituent on Tracheal Smooth Muscle

*O. basilicum* has been revealed to possess bronchodilatory and relaxant effects. Macerated hydroethanolic extract of *O. basilicum* leaf (0.75, 1.50, and 3.00 mg/ml) meaningfully decreased the tracheal smooth muscle (TSM) contractile tone induced by methacholine, KCl, and airways inflammation in a dose-dependent manner (Pérez-Guerrero et al., 1997; Eftekhar et al., 2019a).

It seems that if *O. basilicum* extracts affected potassium channels, they should not have any effect on the TSM contracted by KCl, whereas the extract should affect TSM when contracted by methacholine. While KCl acts on the calcium channels (Pérez-Guerrero et al., 1997) and calcium channel blockers showed a bronchodilatory effect (Mccaig and De Jonckheere, 1993; Miyahara et al., 1993; Janbaz et al., 2014), no calcium channel blocking effects for this extract were shown (S. S. Athari et al., 2016).

The concentration-dependent relaxant effects of four cumulative concentrations of Soxhlet and macerated extracts of *O. basilicum* (0.25, 0.5, 0.75, and 1.0 W/V) on the contractile tone of tracheal smooth muscle, induced by methacholine and KCl in guinea pigs, were found to be comparable to those of theophylline (Boskabady et al., 2005).

Linalool and eugenol, the constituents of *O. basilicum*, were also shown to have a relaxing effect on TSM or a bronchodilator effect similar to the plant itself (Shabir et al., 2014; Silveira et al., 2017). The relaxation effects of eugenol and linalool (100 mM) on TSM in the absence and presence of three inhibitors indicated that the main mechanism responsible for the relaxant effect of linalool is its inhibitory effect on cyclooxygenase signaling cascades, and that for eugenol is its ROS inhibitory effect.

It was shown that linalool fully recovered the electromechanical-induced contraction in TSM with intact and denuded epithelium, but the effect was lower in TSM with intact epithelium. Linalool inhibits the calcium and barium influx curves. This study showed that the relaxant effect of linalool was mediated by the blocking effect on calcium influx through voltage-dependent channels. These results also indicate that linalool and eugenol are responsible for the relaxant effect of *O. basilicum* on TSM.

Other likely mechanisms for bronchodilatory effects of *O. basilicum* are stimulating β-adrenergic but blocking histamine H1 receptors, anticholinergic function, inducing inhibitory nerves other than cholinergic system (NANC), or prevention of stimulatory NANC, methylxanthine similar activity, as well as phosphodiesterase inhibitory effect (S. S. Athari et al., 2016).

These findings showed the relaxant effect of essential oils of *O. basilicum* on TSM which was suggested to be due to the presence of linalool and eugenol. The possible mechanisms of the relaxant effect of *O. basilicum* and its constituents are stimulating β-adrenergic, inhibiting histamine H_1_ and muscarinic receptors, blocking calcium channels, methylxanthine similar activity, and phosphodiesterase inhibitory effect. Therefore, the plant and its constituents could be used as bronchodilatory agents in obstructive pulmonary diseases. A summary on the relaxant effects of *O. basilicum* and its ingredients is presented in Table 2.

**TABLE 2:** Summarized experimental effects of *O. basilicum* on the respiratory system.

Part of plant	Disease model	Brief results	Ref.
Leaf	Bronchospasm	Reduction of the tracheal smooth muscle contractile tone	(Janbaz et al., 2014; Eftekhar et al., 2019b)
Linalool and eugenol	Bronchospasm	Reduction of the tracheal smooth muscle contractile tone	(Shabir et al., 2014; Silveira et al., 2017)
Seed	Asthma	Suppressed gene expression of Th2 cytokines (IL-4, 5, and 13) and mucus discharge in the airways	(S. M. Athari et al., 2018)
Seed	Asthma	Inhibition of the expression of Th2 upstream cytokines including IL-8, 9, 11, 25, 33 and eotaxin and hyperplasia in goblet cell in allergic asthma	Nasaba et al. (2020)
Essence	Allergic asthma	Increase in PLA2, IgE, IL-4, and TP levels and reduction of the IFN-γ/IL-4 level	Eftekhar et al. (2019a)
Seed	COPD	Inhibition of the apoptosis caused by H2O_2_-oxidative stress in respiratory cells	Vardhan (2013)
Apigenin, linalool, and ursolic acid	Bronchitis	Antiviral effect against adenovirus	Amber et al. (2017)
Aerial parts	Lung cancer	Suppressed viability of A549 cells, which might due to the activation of apoptotic signaling and the inhibition of anti-apoptotic cascades	Arshad Qamar et al. (2010)

#### Preventive Effect of *O. basilicum* and Its Main Constituent on Respiratory Disorders

##### Asthma and Allergy

Allergic asthma is a complex illness of the lung characterized by wheezing, coughing, eosinophilia, dyspnea, and lung inflammation (Ying et al., 1999). The bronchial constriction, mucus hyper-secretion, and enhanced airway responsiveness are important properties in asthma pathophysiology (S. S. Athari et al., 2017). Interleukin (IL)-4, IL-13, IL-5, and Th2 cytokines have a significant role in asthma development (Mould et al., 2000).

Th1/Th2 imbalance toward increasing Th2 activity appears in asthma pathogenesis. Cytokines released from Th2 cells like IL-5 and IL-4 stimulate IgE secretion from the B cells that play an important role in allergic asthma. Secretion of phospholipase A2 establishes lung inflammation in asthma by arachidonic acid releasing, cytokine diffusion, lysophospholipid production, and the effect of inflammatory and immunologic cells. Medicines currently used for asthma decrease lung inflammation and bronchospasm; however, following termination of treatment, symptoms recur. For a long time, asthma has been treated using herbal medicines in the Middle East, China, and Europe.

Treatment of BALB/c mice with asthma induced by the ovalbumin (OVA, 20 μg, i.p) with *O. basilicum* (orally, on days 25, 27, and 29) suppressed Th2 cytokine gene expression (IL-4, 5, and 13) (S. M. Athari et al., 2018). Treatment with *O. basilicum* decreased goblet cell hyperplasia and mucus hyper-secretion in the asthmatic lung segments (S. M. Athari et al., 2018). In this study, Athari et al. described that *O. basilicum* might increase Th1 cytokine gene expression and Th1/Th2 cytokine balance. This balance is essential for the management and treatment of allergic asthma. Thus, immunomodulatory and anti-inflammatory effects of *O. basilicum* in allergic asthma were shown, and it was suggested that this plant could be considered for the treatment of asthma (S. M. Athari et al., 2018). In another study, it has been reported that oral administration of *O. basilicum* seed extract (on days 25, 27, and 29 from 30 days of sensitization period) inhibits the expression of Th2 upstream cytokines such as IL-8, IL-9, IL-11, IL-25, IL-33, and eotaxin and hyperplasia in goblet cell in an animal model of OVA-induced allergic asthma. These findings also revealed that therapeutic effects of the plant in asthma were induced by the inhibition of the Th2 expression and goblet cell hyperplasia (Nasaba et al., 2020).

In a rat model of asthma induced by OVA, *O. basilicum* hydroethanolic extract (0.75, 1.50, and 3.00 mg/ml) in drinking water, for 21 days during the sensitization period, reduced IL-4, PLA2, IgE, and total protein (TP) levels but enhanced the IFN-γ/IL-4 ratio in a dose-dependent manner. Lung pathological changes in asthmatic animals were also improved by the plant extract. The results of this study suggest the therapeutic potential of *O. basilicum* in asthma by improving lung pathological, inflammatory, and immunological changes in sensitized rats (Eftekhar et al., 2019b). In rats sensitized to OVA, treatment with the extract of *O. basilicum* (0.75, 1.5, and 3.0 mg/ml), rosmarinic acid (0.125, 0.25, and 0.5 mg/ml) administered in the animals’ drinking water, and dexamethasone (1.25 μg/ml) decreased total and differential white blood cells (WBC) and improved serum levels of antioxidant and oxidant markers; these effects were comparable with the effect of dexamethasone. In another study with a similar methodology, tracheal responsiveness to OVA and methacholine; total WBC count; percentages of monocytes, eosinophils, and neutrophils; and levels of oxidant markers in the BALF were markedly reduced, but antioxidant markers were enhanced due to treatment with the extract of *O. basilicum*; these effects were similar to the effect of dexamethasone in sensitized rats. Treatment of asthmatic rats induced by OVA with rosmarinic acid (0.125, 0.25, and 0.5 mg/ml) also reduced the tracheal responsiveness to OVA and methacholine; total and differential WBC count, and levels of oxidant markers in the BALF increased, but levels of antioxidant markers decreased, which were similar to dexamethasone effects. In asthmatic rats, rosmarinic acid (0.125, 0.250, and 0.500 mg/ml) decreased the levels of IL-4, PLA2, IgE, and total protein, and increased the IFN-/IL-4 ratio in the BALF. Lung pathological changes induced by OVA were improved by rosmarinic acid treatment (Shakeri et al., 2019). The findings of these works suggest a therapeutic effect for the extract of *O. basilicum* and its constituent, rosmarinic acid, on asthma (Eftekhar et al., 2018a; Eftekhar et al., 2018b; Eftekhar et al., 2019a).

*O. basilicum* and its constituents, including linalool, eugenol, and more than 5 oxygenated monoterpene derivatives, have an anti-inflammatory effect (Okoye et al., 2014). *O. basilicum* terpenoids have been reported to suppress nuclear factor kappa B (NF-κβ) signaling and nitric oxide (NO) production, thus producing anti-inflammatory effects. NO is an inflammatory molecule that plays a role in immunoregulation. NO inhibitors are of therapeutic importance in preventing pathological conditions caused by inflammation. On the other hand, the inhibition of the c-Jun subunit of the activator protein 1 (AP-1) suppresses transcription of inflammatory genes, leading to the inhibition of the inflammatory pathways. Anti-inflammatory effect of the *Ocimum* genus was shown to be induced through inhibition of pro-inflammatory cytokines (Kapewangolo et al., 2015). Also, the fixed oils of the *Ocimum* genus may have the potential to inhibit the cyclooxygenase and lipoxygenase pathways. Agents that affect the pathways of the arachidonic acid metabolism would be of great value in inflammatory conditions such as asthma (Singh et al., 1996). Iranian *O. basilicum* is used for improvement of pharynx congestion, fever, and stomachache.

Treatment with *O. basilicum* suppressed Th2 cytokines’ (IL-4, 5, IL-8, IL-9, IL-11, 13 IL-25, and IL-33) gene expression; oxidant markers; total and differential WBC both in the blood and the BALF; reduced PLA2, IgE, and TP; alleviated mucus hyper-secretion and lung pathological changes (including goblet cell hyperplasia and airway responsiveness); and enhanced Th1 cytokine gene expression and Th1/Th2 balance but increased antioxidant marker levels in an animal model of asthma. Rosmarinic acid, a constituent of the plant, also showed similar effects as the plant. *O. basilicum* terpenoids also suppressed NF-κβ signaling and the NO level. Therefore, since *O. basilicum* and its constituents suppressed lung inflammation as the main characteristic of asthma, they could be considered candidates for the prevention of asthma.

##### Chronic Obstructive Pulmonary Disease

COPD is a type of obstructive respiratory illness categorized by long-term breathing complications and poor airflow (Macnee, 2005). The main signs of the disease are cough with sputum production and shortness of breath (Agusti et al., 2010) which deteriorates over time (Mannino and Buist, 2007).

There is limited information about the effect of *O. basilicum* on COPDs. However, it is reported that *O. basilicum*, *O. gratissimum*, and *O. tenuiflorum* are the three herbal plants of the *Ocimum* genus which inhibit apoptosis caused by H_2_O_2_-oxidative stress in respiratory cells, showing potential therapeutic effects against COPDs (Vardhan, 2013).

The average cell viability was meaningfully different between the groups treated with the three plants as follows: *O. tenuiflorum* (holy basil) (90.25%), *O. gratissimum* (wild basil) (75.49%), and *O. basilicum* (sweet basil) (74.28%). Therefore, *O. tenuiflorum* had superior effects than *O. gratissimum* and *O. basilicum* which showed similar potential.

*In vitro*, linalool was reported to inhibit endotoxin-induced production of TNF-α and IL-1β in RAW264.7 macrophage, and TNF-α, IL-1β, nitric oxide, and prostaglandin E2 in a murine microglial cell line BV2 (Lee et al., 2018). Linalool also attenuated lung inflammation in COPDs, inhibited the infiltration of inflammatory cells, and reduced IL-1β, IL-6, IL-8, TNF-α, and monocyte chemoattractant protein (MCP)-1 production. Also, linalool inhibited lung myeloperoxidase (MPO) activity and pathological changes. Linalool suppressed NF-κB activation in a dose-dependent manner. Linalool also inhibits cigarette smoke–induced lung inflammation by inhibiting NF-κB activation. Besides, eugenol causes the inhibition of pro-inflammatory mediators such as COX-2, NF-κB, IL-6, leukotriene C4, and 5-LOX (De Araújo Lopes et al., 2018).

Therefore, this plant could be of therapeutic value on COPDs due to its antioxidant constituents. The effect of *O. basilicum* and its constituents on asthma and COPDs shown by experimental studies is presented in Table 2.

##### Bronchitis

Bronchitis is a lung infection primarily caused by the rhinovirus, influenza virus, coronavirus, adenovirus, and other viruses (Amber et al., 2017) in which the mucous membrane of the bronchial tracts develops inflammation and causes defect in oxygen transfer from the trachea to the lungs (Pierangeli et al., 2007).

The bioactive ingredients of *O. basilicum* have been described to prevent different viral infections. Three types of secondary metabolites including linalool, apigenin, and ursolic acid have been described against various types of human adenovirus complications in bronchitis. Among these, ursolic acid displayed extreme inhibition (50%) of adenovirus. Triterpenoids such as saponin suppressed the viral activity by inhibiting the DNA and viral protein capsid synthesis (Amber et al., 2017).

##### Lung Cancer

Lung cancer, or lung carcinoma, is a malignant respiratory tumor categorized by uncontrolled cell growth in lung tissue (Barta et al., 2019). These growths can be extended by the progression of metastasis into close tissue or other parts of the body (Ridge et al., 2013). Two chief types of such cancers are small-cell lung carcinoma and non–small-cell lung carcinoma. The most common signs of lung cancer are cough, shortness of breath, chest pain, and weight loss (Baumann et al., 2009; Makinoshima et al., 2012).

In a study, Arshad Qamar et al. (2010) described the antiproliferative activity of extract of aerial parts of *O. basilicum* (40 mg/ml) with dimethyl sulfoxide (DMSO) in MCF-7 cells which might partially be due to the impacts of ursolic acid on microtubules and F-actin. Ursolic acid (40, 60, and 100 μM) induced F-actin aggregation after 24 h of incubation. This may be explained by either inhibition of depolymerization of actin filaments or stabilization and promotion of actin polymerization.

The molecular mechanism of ursolic acid–induced apoptosis may possibly involve the release of apoptogenic molecules, in particular both AIF and Endo G, from mitochondria in NCI-H292 human lung cancer cells (Chen et al., 2019).

Also, the aqueous extract of *O. gratissimum* suppressed the viability of A549 cells, which might be due to the inhibition of antiapoptotic cascades and activation of apoptotic signaling, suggesting that the aqueous *O. gratissimum* extract might be helpful in lung carcinoma management (Arshad Qamar et al., 2010).

*O. basilicum*, *O. gratissimum*, and *O. tenuiflorum* inhibited apoptosis caused by H_2_O_2_-oxidative stress in respiratory cells, indicating their potential as a possible medication for COPD. *O. basilicum* constituents including linalool, apigenin, and ursolic acid can prevent different viral infections including human adenovirus bronchitis. The antiproliferative effect of *O. basilicum* in MCF-7 cells and lung carcinoma was shown to be partially due to the impacts of ursolic acid. Therefore, this plant could be of therapeutic value against COPDs, bronchitis, and lung cancer. The effect of *O. basilicum* and its ingredient on bronchitis and lung cancer reported by experimental studies is presented in Table 2.

### The Effects of *O. basilicum* on Respiratory Disorders: Clinical Evidence

#### Pulmonary Aspergillosis

*Aspergillus* spp. frequently causes human bronchopulmonary infections, mostly in immunocompromised patients (Hedayati et al., 2016; Taghizadeh-Armaki et al., 2017a; Pinto et al., 2018; Omran et al., 2019). It causes a broad spectrum of infections including allergic bronchopulmonary aspergillosis, chronic pulmonary aspergillosis, aspergilloma, and invasive aspergillosis, the most serious clinical issue (Nasri et al., 2015; Taghizadeh-Armaki et al., 2017b).

Aspergillosis comprises various diseases produced by the members of the *Aspergillus* genus such as *A. flavus*, *A. fumigatus*, *A. niger*, and *A. terreus* that are the common species accountable for infections (Bahkali et al., 2015).

Bahkali et al. (2015) reported that *O. basilicum* (20 mg/ml for 7 days) generally prevented the hyphae growth and spore germination of *A. fumigatus* and *A. terreus*. Also, this extract exhibited a strong antifungal property *versus* the hyphae growth and spore germination of *A. versicolor* and *A. flavus*.

Also, it has been reported that essential oil extracted from *O. basilicum* leaves (0.4 μl/ml) has strong antifungal activity *versus* several aflatoxigenic fungi that contaminate foods, including *A. flavus*, *Fusarium oxysporum*, *Alternaria alternata*, *A. fumigatus*, *Curvularia lunata*, *Penicillium italicum*, *A. niger*, and *F. nivale* (Singh et al., 2011).

Linalool can interfere in the biosynthesis of the cell wall and/or increase the ionic permeability of the fungal cell membrane. It may be regarded as an agent capable of controlling fungal infections; for instance, antifungal activity of linalool in cases of *Candida* spp. isolated from individuals with oral candidiasis was shown (Dias et al., 2017).

#### Lung Tuberculosis

Tuberculosis is caused by mycobacterial strains; for example, *Mycobacterium tuberculosis*, *M. africanum*, and *M. bovis*, identified cooperatively as the *M. tuberculosis* complex (Haouat et al., 2012).

It has been indicated that 80% methanolic extracts of *O. basilicum* seed (75 μg/ml solvent) exhibit promising antimycobacterial activity *versus M. tuberculosis* and *M. bovis* strains (Gemechu et al., 2013). This might be due to *O. basilicum* bioactive ingredients such as saponins, tannins, alkaloids, flavonoids, and polyphenols that exist in the extract (Arya, 2011; Radji et al., 2015). Although the antimicrobial potential of *O. basilicum* had been described on several other human pathogenic bacteria (Nibret and Wink, 2010; Hussien et al., 2011; Sharaf et al., 2021), no study assessed its effect on *Mycobacterium tuberculosis*.

Another study reported that inhibition of *M. tuberculosis* by pure *O. basilicum* aerial parts’ extract (6.25 μg/ml solvent) supports the usage of this herb as a complementary medicine for improvement of the signs of respiratory tuberculosis (Siddiqui et al., 2012).

The essential oil of *O. basilicum* revealed antifungal and antibacterial properties (Dube et al., 1989; Siddiqui et al., 2007). *O. basilicum* extracts and vital oils can also be used to manage lung infections. *O. basilicum* essential oil (16 μg/ml solvent) prevents arachidonic acid metabolism including cyclooxygenase and lipoxygenase pathways. The watery essence of *O. basilicum* suppressed giant cell generation in Molt-4 cells with and without HIV-1 infection, indicating its inhibitory activity against HIV-1 reverse transcriptase (Yamasaki et al., 1998).

*O. basilicum* showed a strong antifungal effect including suppression of the hyphae growth and spore germination of *A. versicolor*, *A. flavus*, and other fungi. The antimycobacterial effect of *O. basilicum* on *M. tuberculosis* and *M. bovis* strains was also shown. Therefore, *O. basilicum* and its constituents could be used for the management of lung infection and lung cancer.

Antibacterial effects of linalool have been shown in several works. Linalool destroys the membrane integrity and increases membrane permeability. Also, considering membrane potential, linalool caused cell membrane depolarization and irregular cell metabolism activity. A study regarding antibacterial activity of linalool against *Pseudomonas aeruginosa* showed respiratory chain dehydrogenase inhibition that finally led to cell death. Table 3 illustrates various clinical effects of *O. basilicum* and its ingredients in respiratory disorders.

**TABLE 3:** Summarized clinical effects of *O. basilicum* on respiratory diseases.

Part of plant	Disease model	Brief results	Ref.
Leaf	Aspergillosis	Very strong antifungal susceptibility *versus* the hyphae growth and spore germination of *A. versicolor* and *A. flavus*	Bahkali et al. (2015)
Essential oil	Aspergillosis	Strong antifungal activity *versus* several aflatoxigenic fungi	(A. K. Singh et al., 2011)
Seed	Lung tuberculosis	Antimycobacterial susceptibility *versus M. tuberculosis* and *M. bovis* strains	Gemechu et al. (2013)
Aerial parts	Lung tuberculosis	Antimycobacterial susceptibility *versus M. tuberculosis*	Siddiqui et al. (2012)
Oil essence	Lung infection	Obstruction of arachidonic acid metabolism, and cyclooxygenase and lipoxygenase paths as well	Yamasaki et al. (1998)

## Toxicity and Safety of *O. basilicum*

Acute toxicity of hydroalcoholic extract of *O. basilicum* (50, 500, 1,000, and 2000 mg/kg, orally) has been evaluated for 14 days in Wistar rats, and its sub-chronic toxicity was examined by administering 50, 200, and 500 mg/kg of extract for 45 days. Various variables including body weight changes, food and water consumption, clinical signs, hematological and biochemical parameters, as well as mortality, were monitored during the study period. At the end of the study, weight of the liver and left kidney and liver, histological markers were also assessed. The LD50 of *O. basilicum* in the acute study was higher than 5 mg/kg. In the sub-chronic study, a reduction in the hematocrit, platelets, and RBC was observed, but no adverse effects on serum parameters and in other variables were seen. The results showed no death or any abnormal dose-dependent changes in biochemical and liver histopathological parameters in the sub-chronic oral administration of *O. basilicum* in Wistar rats, but several hematological changes had occurred. The results indicated the negligible risk of oral *O. basilicum* consumption (Rasekh et al., 2012).

In another study, acute and subacute toxicity of *O. basilicum* oils in rats was studied following a 14-day gavage administration in Wistar rats, and the animal’s general behavior and survival were assessed daily, and their stomach and liver histological analyses were done at the end of the study. Doses higher than 1,500 mg/kg of the oils significantly changed the stomach and liver histology of rats, but no adverse effect was observed. Therefore, the results indicated that the oil of *O. basilicum* can be considered safe for humans at recommended doses (Fandohan et al., 2008).

Acute toxicity of *O. gratissimum* (*Ocimum* oil), the other plant of this family, was examined by oral and intraperitoneal administration of its graded doses as a 4% v/v (LD50 and LD100). Sub-chronic toxicity of the oil was evaluated by the administration of three graded sub-lethal doses for 30 days, and organs and blood samples were taken at the end of the study. In the acute toxicity study, a dose-dependent sedative effect of *Ocimum* oil was seen in both mice and rats, and the sub-chronic test was done only in rats. There were significant weight changes in the testes, hearts, kidneys, intestines, and lungs as well as significant changes in blood biochemical and hematological variables. Therefore, *Ocimum* oil has toxic potentialities at higher doses and might be better tolerated when administered orally for systemic delivery (Orafidiya et al., 2004).

*Ocimum* (2000, 4,000, 6,000, and 8,000 mg/kg, orally) was administered to Swiss mice for acute toxicity assessment, and death and body weight changes were monitored for 7 days. However, for subacute toxicity assessment, rats were treated with three doses of 250, 500, and 1,000 mg/kg/day for 42 consecutive days, and biochemical and hematological variables, and organs’ histology were assessed at the end of the study. The extract up to 8,000 mg/kg did not show mortality or significant changes in general behavior. Body and organ weights, feeding habits, or behavior also did not change in the sub-chronic study, and no toxicity was observed in hematological or blood biochemistry variables or organs’ histology. These results showed non-toxic effects of acute and sub-chronic intake of leaf aqueous extract of *Ocimum* (Tan et al., 2008).

## Discussion

Various studies showed the relaxant effects of *O. basilicum* and its main constituents including linalool and eugenol, and showed the bronchodilatory effects of the plant and its components against obstructive pulmonary diseases with various possible mechanisms.

The preventive effects of *O. basilicum* and its constituents such as linalool, eugenol, rosmarinic acid, and terpenoids against asthma were also reported, and several possible mechanisms were shown. The extracts of the plant and its constituents decreased mucus hyper-secretion and goblet cell hyperplasia, inhibited the cyclooxygenase and lipoxygenase pathways, as well as suppressed nuclear factor kappa B (NF-κβ) signaling and nitric oxide (NO) generation in a mouse model of asthma induced by OVA. Lung pathological changes and airway responsiveness to OVA and methacholine were also reduced by the plant and its components in a rat model of asthma. *O. basilicum* and its derivatives also reduced TNF-α, IL-1β, and IL-2, and inducible nitric oxide synthase (iNOS). In addition, the gene expression of Th2 cytokines (IL-4, 5, and 13) was suppressed and Th1/Th2 cytokine balance was increased in asthmatic animals. All these findings strongly suggest the possible therapeutic effect of the plant and its constituents on asthma. *O. basilicum* and its constituents, and other species from *Ocimum* genus, showed potent antioxidant and anti-inflammatory effects which could be considered for the treatment of COPDs.

The extract of *O. basilicum* prevents MCF-7 cell proliferation partially due to the effects of ursolic acid on microtubules and F-actin. The aqueous extract of *O. gratissimum* also suppressed the viability of A549 cells through apoptotic signaling activation and antiapoptotic cascade inhibition. Therefore, the plant and its compounds could be used as an anticancer therapy in lung cancers.

The preventive effect of *O. basilicum* on germination of *A. fumigatus* and *A. terreus* as well as its antifungal property on the hyphae growth and spore germination of *A. versicolor* and *A. flavus*, and the antifungal activity of the plant on several aflatoxigenic fungi and foods contaminated with *A. flavus*, *Fusarium oxysporum*, *Alternaria alternata*, *A. fumigatus*, *Curvularia lunata*, *Penicillium italicum*, *A. niger*, and *F. nivale* were shown; these effects suggested the effect of this plant on pulmonary infections and aspergillosis. The extracts of *O. basilicum* seed showed antimycobacterial activity against *M. tuberculosis* due to its ingredients including tannins, saponins, alkaloids, flavonoids, and polyphenols. The inhibition of *M. tuberculosis* by *O. basilicum* aerial part extract was also shown. These findings suggest the effect of this plant as a complementary medicine for the treatment of respiratory tuberculosis.

The studies of the plant safety also showed a mild toxicity only when very high doses of the plant were tested. Therefore, *O. basilicum* preparations are safe when usual doses are used.

## Conclusion

The current review showed the therapeutic effects of *O. basilicum* and its ingredients on various respiratory diseases. The findings of the reviewed articles revealed both relieving and prevention of the plant preparations on obstructive pulmonary diseases including COPDs and asthma. The preventive effects of *O. basilicum* and its main constituents on other respiratory disorders such as bronchitis, aspergillosis, tuberculosis, and lung cancer were also indicated.

There are limited numbers of clinical studies regarding the effects of the plant and its constituents on respiratory disorders. From the results of the reviewed articles, the average dose of the plant required for the treatment of respiratory disorders was estimated to be 10 mg/kg/day. In addition, the results of the reviewed articles indicate that *O. basilicum* and its constituents can increase the quality of life of patients with respiratory disorders both as a reliever and preventive medication, and could show synergetic effect with the currently used chemical drugs.

**FIGURE 4:**
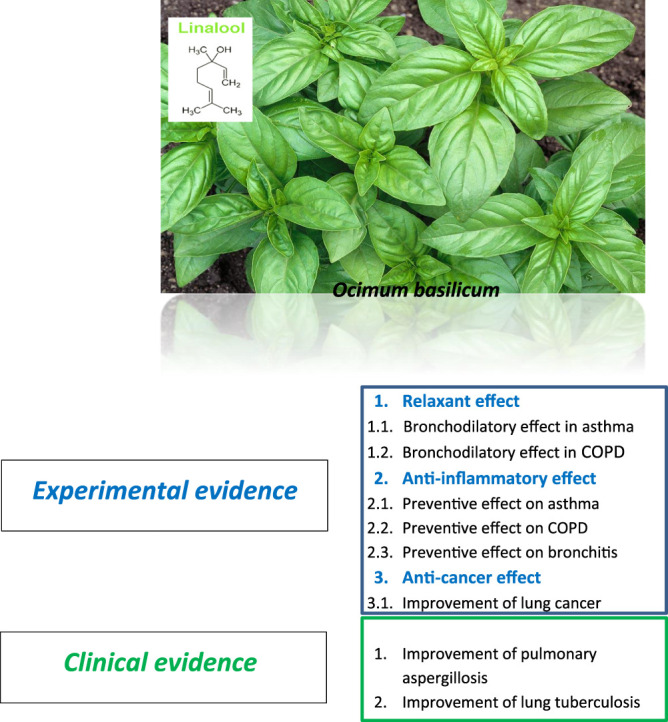
Summary of *O. basilicum* effect on respiratory disorders.

However, further experimental works are needed on the effect of the plant and its constituents on some respiratory disorders such as COPDs, bronchitis, and lung cancer. Before the plant and its constituents could be used in clinical practice, further clinical trials are needed to examine these effects. The exact doses of *O. basilicum* preparation for the treatment of various respiratory diseases should also be determined in further studies. Whether the plant and its derivatives alone could be used to treat respiratory diseases should be clarified in further studies. Figure 4 summarizes the *O. basilicum* effect on respiratory disorders.
